# Genome sequencing of gut symbiotic *Bacillus velezensis* LC1 for bioethanol production from bamboo shoots

**DOI:** 10.1186/s13068-020-1671-9

**Published:** 2020-02-28

**Authors:** Yuanqiu Li, Lu Lei, Li Zheng, Ximeng Xiao, Hao Tang, Chaobing Luo

**Affiliations:** Bamboo Diseases and Pests Control and Resources Development Key Laboratory of Sichuan Province, Leshan Normal University, No. 778, Binhe Road, Central District, Leshan, 614000 China

**Keywords:** *Bacillus velezensis* LC1, Carbohydrate-active enzyme, Bamboo shoot, Cellulose, Ethanol

## Abstract

**Background:**

Bamboo, a lignocellulosic feedstock, is considered as a potentially excellent raw material and evaluated for lignocellulose degradation and bioethanol production, with a focus on using physical and chemical pre-treatment. However, studies reporting the biodegradation of bamboo lignocellulose using microbes such as bacteria and fungi are scarce.

**Results:**

In the present study, *Bacillus velezensis* LC1 was isolated from *Cyrtotrachelus buqueti*, in which the symbiotic bacteria exhibited lignocellulose degradation ability and cellulase activities. We performed genome sequencing of *B. velezensis* LC1, which has a 3929,782-bp ring chromosome and 46.5% GC content. The total gene length was 3,502,596 bp using gene prediction, and the GC contents were 47.29% and 40.04% in the gene and intergene regions, respectively. The genome contains 4018 coding DNA sequences, and all have been assigned predicted functions. Carbohydrate-active enzyme annotation identified 136 genes annotated to CAZy families, including GH, GTs, CEs, PLs, AAs and CBMs. Genes involved in lignocellulose degradation were identified. After a 6-day treatment, the bamboo shoot cellulose degradation efficiency reached 39.32%, and the hydrolysate was subjected to ethanol fermentation with *Saccharomyces cerevisiae* and *Escherichia coli* KO11, yielding 7.2 g/L of ethanol at 96 h.

**Conclusions:**

These findings provide an insight for *B. velezensis* strains in converting lignocellulose into ethanol. *B. velezensis* LC1, a symbiotic bacteria, can potentially degrade bamboo lignocellulose components and further transformation to ethanol, and expand the bamboo lignocellulosic bioethanol production.

## Background

Lignocellulose, a widely distributed, renewable and enormous biomass resource, is one of the most important raw materials for bioethanol production [1]. Bamboo, a lignocellulosic feedstock, is a regenerated biomass material with abundant resources, short growth cycle, high yield and similar chemical composition as wood, and it is considered as a potentially excellent raw material [2, 3]. Many studies have evaluated bamboo lignocellulose degradation and bioethanol production, with a focus on using physical and chemical pre-treatment [4, 5]. However, studies reporting the biodegradation of bamboo lignocellulose using microbes such as bacteria and fungi are scarce.

Lignocellulose hydrolysis, especially cellulose degradation, remains a considerable challenge in lignocellulosic bioethanol production [6]. In nature, numerous examples for lignocellulose degradation are present; of these, phytophagous insects are considered the most notable. In these insects, intestinal symbiotic microbes played important roles in lignocellulose degradation [7]. Therefore, the intestines of phytophagous insects were considered as important locations for isolating lignocellulolytic microbes [8].

Microbial degradation of lignocellulose is a green biological refining method with advantages over physical and chemical methods [9]. The bacterial genus *Bacillus* is an excellent degrader that exhibits various abilities for degrading lignocellulose biomass, including cellulose, hemicellulose and lignin [10, 11]. Furthermore, genome sequencing, considered an efficient method for investigation of function, has been utilized in lignocellulose degradation research. However, the lignocellulose degradation of *Bacillus* is still unclear [12]. Dunlap et al. [13] reported that *B. oryzicola* and *B. methylotrophicus*, were classified into the *B. velezensis* group. Recently, the complete genome and genes associated with lignocellulose degradation of several *B. velezensis* strains were sequenced and are enriched in the genome [14–16]. However, its potential application in converting lignocellulose into bioethanol has received little attention.

In the present study, we isolated an endophytic bacteria from the gut of *Cyrtotrachelus buqueti* that showed a bamboo lignocellulose-degrading ability [17] and sequenced the whole genome of the bacteria *B. velezensis* LC1, determined the cellulase activities and analysed the ethanol production of bamboo shoot. CAZy genes involved in degradation of lignocellulose were identified through genomic analysis. The chemical changes of the cell wall components were investigated, as well as the hydrolytic and ethanol-fermenting properties of bamboo shoots.

## Results and discussion

### Identification and cellulose-degrading potential of *Bacillus velezensis* LC1

**Fig. 1:**
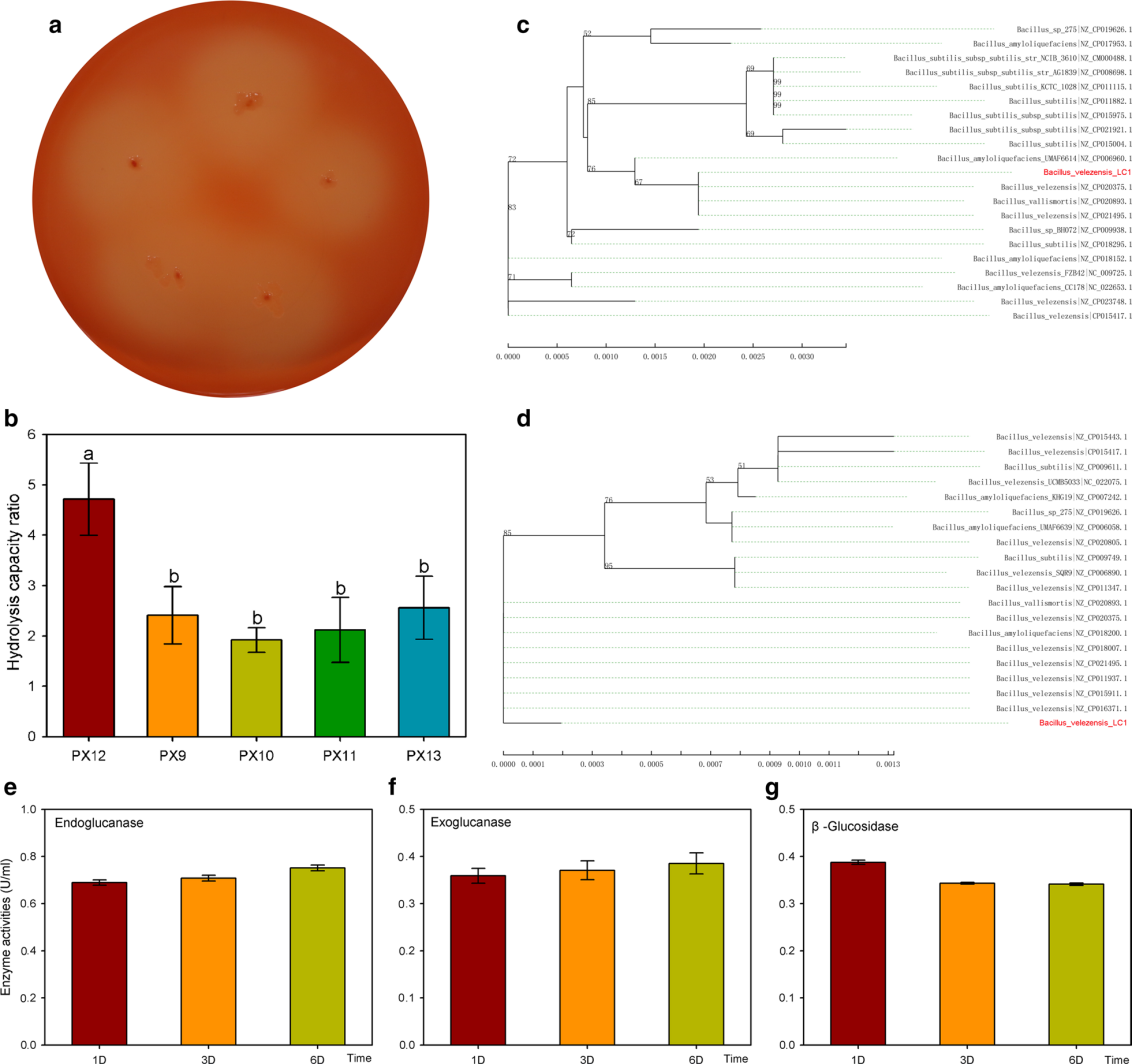
Isolation, identification and enzyme activities of *Bacillus velezensis* LC1. **a** Cellulolytic activities of PX12 cultured on carboxymethyl cellulose agar with Congo red stain. **b** Cellulose hydrolysis capacity ratio of the isolates PX9, PX10, PX11, PX12 and PX13. **c** Phylogenetic analysis of 16S rRNA sequences among *B. velezensis* LC1 and the closest Bacillus BLAST hits. **d** Phylogenetic analysis of house-keeping gene sequences among *B. velezensis* LC1 and the closest *Bacillus* BLAST hits. **e** Endoglucanase activity during the culture for 1st day, 3rd day and 6th day. **f** Exoglucanase activity during the culture for 1st day, 3rd day and 6th day. **g** β-glucosidase activity during the culture for 1st day, 3rd day and 6th day. The different normal letters indicate a significant difference in gene expression at different time points with *p* value at 0.05 level (*n* = 3)

Five cellulolytic strains, including PX9, PX10, PX11, PX12 and PX13, which produced clear zones around the colonies after Congo red staining, were isolated from the intestine of *C. buqueti* on CMC agar. Among the five strains, PX12 exhibited the highest cellulose hydrolysis capacity, with a higher hydrolysis capacity ratio (HCR: 4.71) than PX9 (HCR: 2.41), PX10 (HCR: 1.92), PX11 (HCR: 2.12) or PX13 (HCR: 2.56), as determined using the cellulose hydrolysis assay (Fig. 1a, b; Additional file 1: Figure S1). Based on the HCR ratio, many potent cellulolytic bacteria were previously screened from various regions, such as *Geobacillus* sp. from a hot spring and *Paenibacillus lautus* BHU3 from a landfill site [18, 19]. Similarly, PX12 was considered as a good cellulolytic bacterium and was used for further study.

PX12 was identified and confirmed by *16S rRNA* sequencing. Phylogenetic analysis of the obtained PX12 sequence revealed a 99% resemblance with *B. velezensis* strain JTYP2 (NZ_CP020375.1) (Fig. 1c). Moreover, a house-keeping gene was used for phylogenetic analysis; it showed that the sequence of PX12 had a 98.6% similarity with *B. velezensis* strain S3-1 (NZ_CP016371.1) (Fig. 1d). Overall, the PX12 was identified as *B. velezensis* and was named as *B. velezensis* LC1.

Several *B. velezensis* strains have been noted for their lignocellulose-degrading abilities [14–16]. To investigate the cellulose-degrading ability, *B. velezensis* LC1 was cultured on CMC agar to determine cellulase activities by the dinitrosalicylic acid spectrophotometric (DNS) method for 6 days (Fig. 1e–g) [20]. The cellulase activities of strain LC1 were then determined. The endoglucanase activity was 0.689 ± 0.011 U/ml at day 1 and increased to 0.752 ± 0.013 U/ml at day 6, which was in accordance with the exoglucanase activity (from 0.359 ± 0.016 U/ml to 0.385 ± 0.022 U/ml), whereas the β-glucosidase activity decreased from day 6 to day 1. Previous studies have reported the cellulase activities of other lignocellulolytic *Bacillus* strains. For example, *Bacillus* sp. 275, *Bacillus* sp. R2, *B. velezensis* 157 and *B. velezensis* ZY-1-1 [10, 11, 21, 22] showed similar results as those achieved in our study. This indicated that the strain played a potential role in cellulose degradation.

### Genome sequencing and assembly of *Bacillus velezensis* LC1

**Fig. 2:**
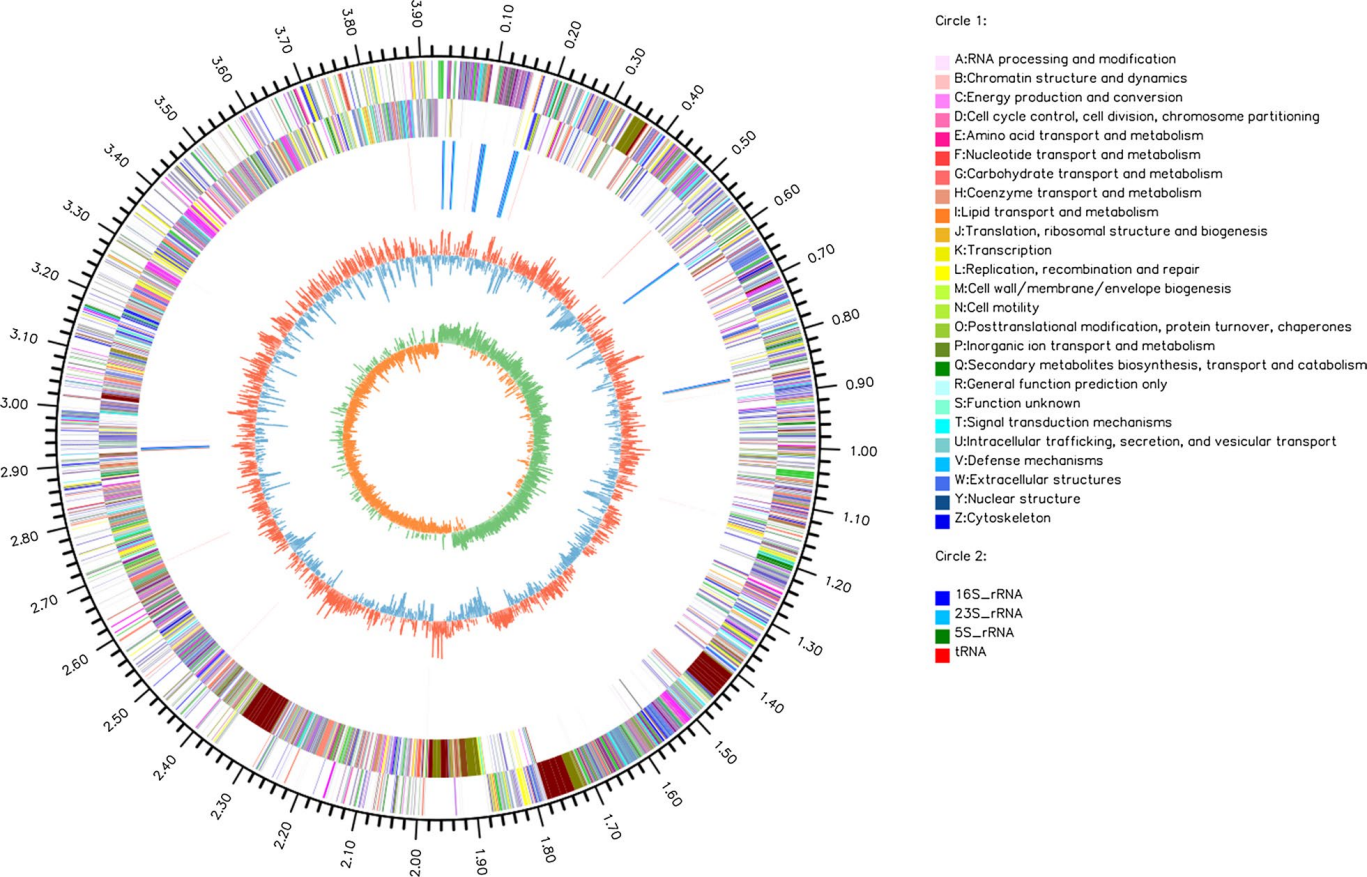
The whole genome of *Bacillus velezensis* LC1. The genome map is composed of seven circles. From the outer circle to inner circle, each circle displays information regarding the genome of (1) forward CDS, (2) reverse CDS, (3) forward COG function classification, (4) reverse COG function classification, (5) nomenclature and locations of predictive secondary metabolite clusters, (6) G+C content and (7) GC skew

The genome contributes to a clear understanding of bacterial decomposition mechanisms of cellulose; thus, the genome of *B. velezensis* LC1 was analysed to decipher the genetic code involved in cellulose degradation. The complete genome sequence of *B. velezensis* LC1 was assembled into a ring chromosome with 3,929,782 bp and had a GC content of 46.5% (Fig. 2). A length of 3,502,596-bp genes was found based on gene prediction, and the ratio of gene length/genome was 89.13%. The intergene region/whole genome ratio was 7.19%, and the GC contents of the gene and intergene region were 47.29% and 40.04%, respectively. Furthermore, 4018 CDSs were contained in the genome, and all were assigned functions. CDSs were further annotated in NR, Swiss-Prot, COGs, KEGGs, GO and Pfam, and their numbers were 4018, 3520, 2996, 2186, 2718 and 3315, respectively (Table 1).

**Table 1:** Genome features of *Bacillus velezensis* LC1

Features	Chromosome
Genome size (bp)	3,929,782
G+C content (%)	46.5
GC content in gene region (%)	47.29
GC content in intergenetic region (%)	40.04
Protein-coding genes (CDS)	4018
Gene total len (bp)	3,502,596
Gene/genome (%)	89.13
Intergenetic region len (bp)	427,187
Intergenetic len/genome (%)	10.87
5s rRNA	9
16s rRNA	9
23s rRNA	9
tRNA	86
NR annotation	4018
Swiss-Prot annotation	3520
Pfam annotation	3315
COG annotation	2996
GO annotation	2718
KEGG annotation	2186

### COGs involved in carbohydrate metabolism

**Fig. 3:**
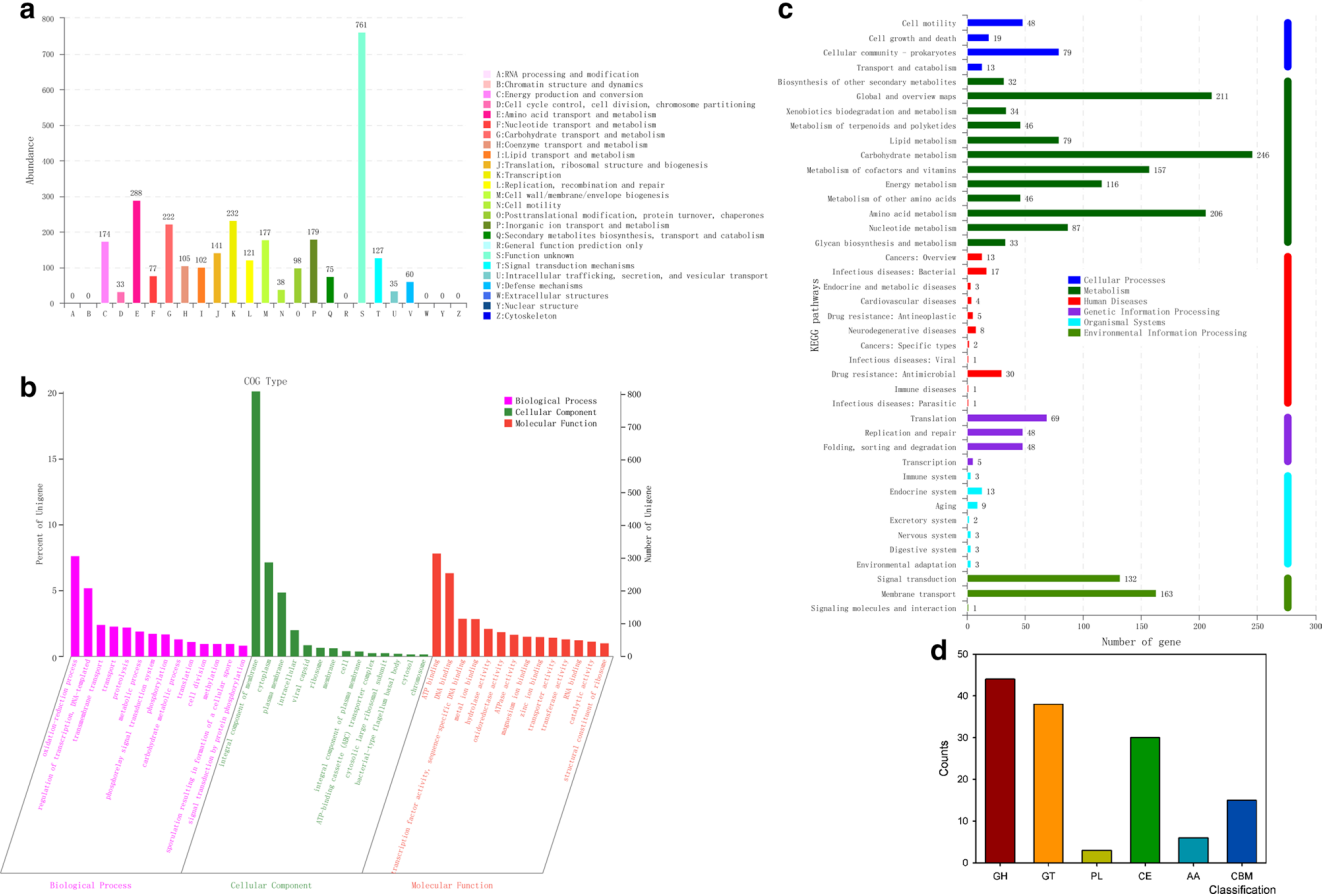
Functional categories of *Bacillus velezensis* LC1. **a** Clusters of Gene Ontology (GO) annotation. **b** Clusters of Orthologous Groups of proteins (COGs) annotation. **c** Clusters of KEGG annotation. **d** Gene count distributions of carbohydrate-active enzyme families. *GH* glycoside hydrolases, *GT* glycosyl transferases, *PL* polysaccharide lyases, *CE* carbohydrate esterases, *CBM* carbohydrate-binding modules, *AA* auxiliary activities

In total, 3046 genes were classified into 2996 COGs, of which carbohydrate transport and metabolism, amino acid transport and metabolism and transcription were the most enriched COGs, which represented 9.46%, 7.62% and 7.29%, respectively (Fig. 3a). To elucidate the function of *B. velezensis* LC1 in cellulose degradation at the genetic level, specific COGs involved in carbohydrate metabolism were analysed. A total of 222 genes were annotated into carbohydrate metabolism, including 130 COGs, of which the most abundant COGs were COG0366 (alpha-amylase), COG0524 (pfkb domain protein), COG2814 (Major facilitator), COG0726 (4-amino-4-deoxy-alpha-l-arabinopyranosyl undecaprenyl phosphate biosynthetic process), COG1263 (PTS System), COG0477 (major facilitator superfamily), COG1455 (pts system), COG1940 (ROK family) and COG2723 (beta-glucosidase) (Additional file 2: Table S1). COG366 encodes an alpha-amylase that acts on a bond between starch and glycogen, hydrolysing polysaccharides into glucose and maltose [23]. COG2814 is involved in cellular transport of some complexes, such as carbohydrates and amino acids. COG0477, a secondary active transporter, helps to catalyse the transport of various substrates [24, 25]. Moreover, other important COGs in carbohydrate metabolism were annotated, e.g. COG0395 was reported to participate in carbohydrate uptake [26] and COG1109 catalysed the conversion of glucosamine-6-phosphate [27]. The high diversity of function annotations indicated that *B. velezensis* LC1 had a potent capability in lignocellulose degradation.

### GO terms annotations

To explain the relevance of the genome of *B. velezensis* LC1, GO analysis was used to categorize genes into three categories according to matches with known sequences. In three categories, molecular function contained most numerous GO terms and gene number (3730), followed by biological process (Gene number: 3025) and cellular component (Gene number: 1637) (Fig. 3b). In molecular function, the most five pathway was ATP binding (GO:0005524; 314 genes), DNA binding (GO:0003677; 254 genes), transcription factor activity (GO:0003700; 115 genes), metal ion binding (GO:0046872; 114 genes) and hydrolase activity (GO:0016787; 84 genes). Oxidation–reduction process (GO:0055114) and regulation of transcription (GO:0006355) were most pathways in the biological process, and integral component of membrane (GO:0016021), cytoplasm (GO:0005737) and plasma membrane (GO:0005886) pathways in cellular component.

Furthermore, we analysed the GOs that are associated with carbohydrate metabolism. We identified 114 GO items associated with carbohydrate metabolism, including GO:0004553 (hydrolase activity that hydrolyses O-glycosyl compounds), GO:0005975 (carbohydrate metabolic processes) and GO:0016787 (hydrolase activity) (Additional file 3: Table S2).

### KEGG annotations

The CDSs of *B. velezensis* LC1 were submitted to KAAS and KEGG pathways to identify metabolism pathways (Additional file 4: Table S3). As shown in Fig. 3c, of the six classification of KEGG pathways, metabolism contained the most numbers of genes, followed by environmental information processing. In KEGG metabolism annotations of *B. velezensis* LC1, carbohydrate metabolism and amino acid metabolism, which are considered its main functions, contained 392 and 285 genes, respectively. For these metabolisms, some pathways were dominant, such as sucrose and starch metabolism (ko00500), glycolysis/gluconeogenesis (ko00010), and amino and nucleotide sugar metabolism (ko00520). Forty-one genes were related to ko00500, and common enzyme endoglucanase (EC.3.2.1.4), present in ko00500, was involved in cellulose degradation (Fig. 3c). Starch and sucrose metabolic pathways occurred in *B. velezensis* LC1, indicating that cellulose could be hydrolysed into cellobiose and ultimately, β-d-glucose. In the genome, 39 genes were found in ko00010, in which d-glucose was phosphorylated into d-glucose-6-phosphate. In addition, ko00010 was linked with other pathways. For example, d-glucose-6-phosphate could be converted to pyruvate, which can be oxidized to acetyl-CoA, having an ability to enter the citrate cycle. Furthermore, ko00520 indicated that glucose from the ko00010 finally entered other pathways under various catalytic reactions. α-d-galactose can be transferred and isomerized in ko00520 and then entered into the ascorbate and aldarate metabolism pathways. Additionally, fructose, 1,4-β-d-xylan, and extracellular mannose were metabolized in ko00520.

The annotation involved in the degradation of lignin or aromatic compounds has also been identified. We identified various enzymes associated with lignin degradation, including oxidoreductase, reductases, dehydrogenases, esterases, thioesterases, transferases and hydrolases. Moreover, 13 monooxygenases, 12 dioxygenases, 2 peroxidases (including one DyP-type peroxidase) and 1 laccase were been identified (Additional file 5: Table S4).

### Carbohydrate-active enzyme (CAZyme) annotation

The bCAN carbohydrate-active enzymes (CAZy) annotation algorithm was used to analyse CAZy annotations to identify genes involved in lignocellulose degradation. The results showed that 136 genes were identified from CAZy families and distributed into five subfamilies. In *B. velezensis* LC1, glycoside hydrolases (GHs), which play key roles in carbohydrates degradation, contained 44 members [28]. Additionally, 38 glycosyl transferases (GTs), 30 carbohydrate esterases (CEs), 3 polysaccharide lyases (PLs), 6 enzymes with auxiliary activities (AAs) and 15 carbohydrate-binding modules (CBMs) were identified (Fig. 3d; Additional file 6: Table S5).

The GH family contained various hydrolases that acted on the glycosidic bond. For example, endoglucanases (EC 3.2.1.4) from *Bacillus* that have cellulose degradation function usually belonged to the GH5 families [29] (Table 2). In the genome, six GH13s were obliged to hydrolyze starch, such as α-amylase, α-glucosidase and α-glycosidase [30]. Four GH4s, three GH1s and one GH16 exhibited potential cellulose degradation because of enzyme function. Three GH32s were found in the genomic annotations, which contained some hydrolases and levanases, thereby revealing their ability to hydrolyse sucrose [31]. Additionally, Four GH43s, two GH51s and one GH30, responsible for xylan degradation, were considered as other important members for hemicellulose degradation (Table 2). GH43 is an important component of xylan degradation system [32]. One GH5, GH30 and GH1 each were annotated as potential β-glucosidases to utilize cellobiose. One GH53, which hydrolyses (1 → 4)-β-d-galactosidic linkages, was reported as an endo-1,4-β-galactosidase [33]. β-mannosidase was classified into GH26, hydrolysing parts of mannan polysaccharides [34]. Additionally, maltose phosphorylase, which belongs to GH65, was reportedly involved in trehalose degradation [35].

**Table 2:** Annotated genes encoding lignocellulose-degrading enzymes of *B. velezensis* LC1, with a focus on enzymes degrading cellulose, hemicellulose and lignin

Classification	Gene ID	Predicted function
Cellulose-related	gene1950	GH5 (endo-1,4-β-glucanase EC 3.2.1.4)
	gene2084	GH30 (glucan endo-1,6-β-glucosidase EC 3.2.1.-)
	gene0865	GH4 (6-phospho-β-glucosidase EC 3.2.1.86)
	gene2256	CH4 (glycerol-3-phosphate dehydrogenase)
	gene2945	GH4 (alpha-glucosidase/alpha-galactosidase)
	gene3871	GH4 (6-phospho-β-glucosidase EC 3.2.1.86)
	gene1280	GH1 (6-phospho-beta-galactosidase)
	gene2085	GH1 (aryl-phospho-beta-d-glucosidase)
	gene3887	GH1 (6-phospho-β-galactosidase EC 3.2.1.85)
	gene3931	GH16 (β-glucanase EC 3.2.1.-)
	gene3442	GH32 (sucrose-6-phosphate hydrolase EC 2.4.1.-)
	gene3822	GH32 (sucrose-6-phosphate hydrolase EC 2.4.1.-)
	gene4083	GH32 (levanase EC 3.2.1.65)
	gene0317	GH13 (α-glucosidase EC 3.2.1.20)
	gene3059	GH13 (α-glucosidase EC 3.2.1.20)
	gene3448	GH13 (α-glucosidase EC 3.2.1.20)
	gene0791	PL1 (pectate lyase EC 4.2.2.-)
	gene3935	PL1 (pectate lyase EC 4.2.2.-)
	gene3515	PL9 (pectate lyase EC 4.2.2.2)
Hemicellulose-related	gene3891	GH26 (β-mannosidase EC3.2.1.78)
	gene3949	GH43 (arabinan endo-1,5-α-l-arabinosidase EC 3.2.1.-)
	gene2795	GH43 (arabinan endo-1,5-α-l-arabinosidase EC 3.2.1.-)
	gene1955	GH43 (arabinoxylan arabinofuranohydrolase EC 3.2.1.-)
	gene1870	GH43 (1,4-β-xylosidase EC 3.2.1.37)
	gene2761	GH51 (α-*N*-arabinofuranosidase EC 3.2.1.55)
	gene2785	GH51 (α-*N*-arabinofuranosidase EC 3.2.1.55)
	gene1954	GH30 (glucuronoxylanase EC 3.2.1.-)
	gene0351	CE 7 (acetylxylan esterase EC 3.1.1.72)
	gene0450	CE 3 (acetylxylan esterase EC 3.1.1.72)
	gene2138	CE 3 (acetylxylan esterase EC 3.1.1.72)
	gene1274	GH53 (arabinogalactan endo-1,4-β-galactosidase EC 3.2.1.89)
Lignin-related	gene1891	AA10 (chitin-binding protein)
	gene2781	AA4 (glycolate oxidase)
	gene0978	AA6 (*p*-benzoquinone reductase)
	gene0576	AA7 (FAD-binding protein)
	gene0946	AA7 (FAD-dependent oxidase)
	gene1182	AA7 (FAD-binding protein)

CEs contributing to the decomposition of xylans were also identified in the genome, including two CE3s, one CE7s, three CE10s, and seven CE4s. CE3 as a potential acetyl xylan esterase enhanced xylan solubilization [36]. The acetylxylan esterase CE7 was considered as a capable xylan-degrading enzyme [37]. CE10 previously exhibited carboxylesterase and xylanase activities involved in hemicellulose degradation [38]. Polysaccharide deacetylases, which play a role in degrading polysaccharides and are classified as a CE4, were also identified. CE4 contained not only highly specific acetylxylan esterases, but also peptidoglycan *N*-deacetylates involved in chitin degradation [39].

Moreover, two PL1s and one PL9 were annotated to degrade pectin. Pectate lyase (EC 4.2.2.2) usually has (1 → 4)-α-d-galacturonan cleavage function, causing oligosaccharides present at the end [40] (Table 2). AA4, AA6, AA7 and AA10 were included in the genome. AA4 included vanillyl-alcohol oxidases, which could transform some phenols [41]. Additionally, AA7 enzymes were involved in biotransformation or detoxification of lignocelluloses [42]. Finally, we proposed a hypothetical cellulose-degrading and ethanol-producing pathway for *B. velezensis* LC1 (Table 3).

**Table 3:** Enzymes in cellulose-degrading and ethanol-producing pathways of *B. velezensis* LC1

Gene ID		Enzymes definitions
1	gene1537; gene2613	PTS system, fructose-specific IIA component [EC:2.7.1.69]
2	gene3441; gene3821	Beta-fructofuranosidase [EC:3.2.1.26]
3	gene0676; gene3887; gene3888	Fructokinase [EC:2.7.1.4]
4	gene3065	Glucose-6-phosphate isomerase [EC:5.3.1.9]
5	gene1004	Phosphoglucomutase [EC:5.4.2.2]
6	gene1974; gene1975; gene3557	UTP-glucose-1-phosphate uridylyltransferase [EC:2.7.7.9]
7	gene1950	Endoglucanase [EC:3.2.1.4]
8	gene2486	Glucokinase [EC:2.7.1.2]
9	gene3065	Glucose-6-phosphate isomerase [EC:5.3.1.9]
10	gene2837	6-Phosphofructokinase 1 [EC:2.7.1.11]
11	gene3722	Fructose-bisphosphate aldolase, class II [EC:4.1.2.13]
12	gene3378	Triosephosphate isomerase (TIM) [EC:5.3.1.1]
13	gene2815; gene3381	Glyceraldehyde 3-phosphate dehydrogenase [EC:1.2.1.12]
14	gene3380	Phosphoglycerate kinase [EC:2.7.2.3]
15	gene3377	2, 3-Bisphosphoglycerate-independent phosphoglycerate mutase [EC:5.4.2.12]
16	gene3377	Enolase [EC:4.2.1.11]
17	gene2837	Pyruvate kinase [EC:2.7.1.40]
18	gene1567	Pyruvate dehydrogenase E1 component alpha subunit [EC:1.2.4.1]
19	gene1568	Pyruvate dehydrogenase E1 component beta subunit [EC:1.2.4.1]
20	gene1570	Pyruvate dehydrogenase E2 component (dihydrolipoamide acetyltransferase) [EC:2.3.1.12]
21	gene0862	Dihydrolipoamide dehydrogenase [EC:1.8.1.4]
22	gene3777	Phosphate acetyltransferase [EC:2.3.1.8]
23	gene2856	Acetate kinase [EC:2.7.2.1]
24	gene0778; gene2879; gene4000	Aldehyde dehydrogenase (NAD +) [EC:1.2.1.3]
25	gene1937; gene2005; gene3037; gene0198; gene0266; gene0591; gene0675; gene0788	Alcohol dehydrogenase [EC:1.1.1.1]

### Comparative genomic analysis of CAZymes with other *B. velezensis* strains

The assembled genome of *B. velezensis* LC1 was compared to the genomes of other 10 *B. velezensis* strains, including, *B. velezensis* S3-1, *B. velezensis* LS69, *B. velezensis* JTYP2, *B. velezensis* DR-08, *B. velezensis* FZB42, *B. velezensis* LPL-K103, *B. velezensis* TB1501, *B. velezensis* UCMB5036, *B. velezensis* LB002 and *B. velezensis* 157. The result showed that GH, GT and PL family numbers were the same in theses strains, while the strain LC1 contained more CE and AA family members and less CBM members (Table 4). The coexistence of these genes suggests that they play important roles in the enzymatic degradation of cellulose and hemicellulose. We consider these degradation enzymes in *B. velezensis* to have potential use for bioethanol production.

**Table 4:** Comparative genomic analysis of CAZymes with other *B. velezensis* strains

Strains	GHs	GT	PL	CE	AA	CBM
*B. velezensis* LC1	43	38	3	30	6	15
*B. velezensis* S3-1	40	36	3	10	1	27
*B. velezensis* LS69	40	36	3	10	1	27
*B. velezensis* JTYP2	39	36	3	10	1	28
*B. velezensis* DR-08	40	36	3	10	1	27
*B. velezensis* FZB42	37	36	3	10	1	21
*B. velezensis* LPL-K103	36	32	3	10	1	29
*B. velezensis* TB1501	40	36	3	10	1	28
*B. velezensis* UCMB5036	40	36	3	10	1	26
*B. velezensis* LB002	42	37	3	10	1	27
*B. velezensis* 157	41	36	3	10	1	29

### Gene expression analysis for *B. velezensis* LC1 cultured in bamboo shoot powder or glucose medium

**Fig. 4:**
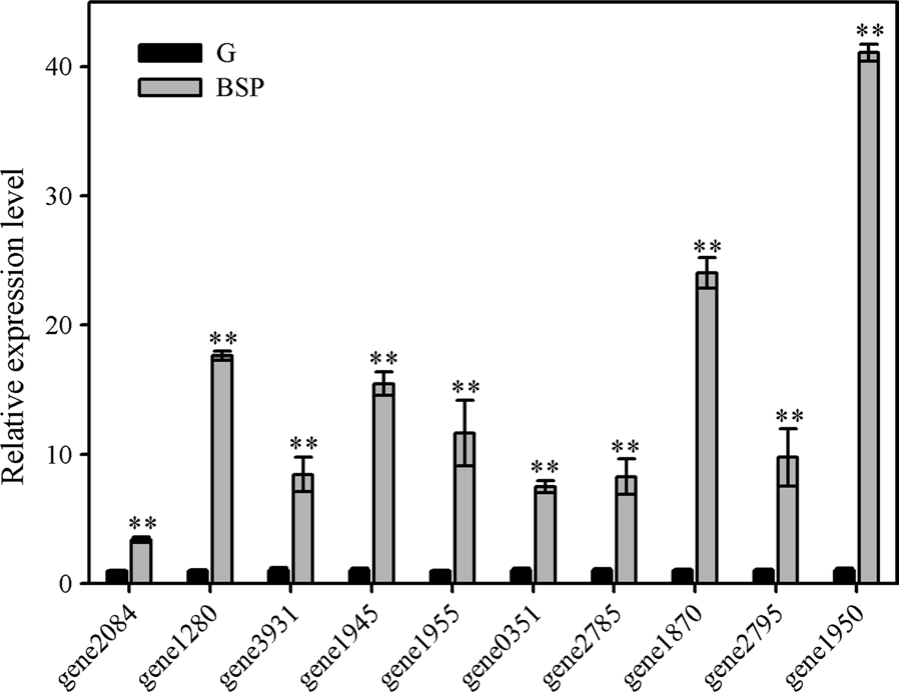
Lignocellulolytic enzyme genes relative expression levels of *B. velezensis* LC1 in the presence of different substrates. *G* glucose substrate, *BSP* bamboo shoot powder substrate. gene1950: endoglucanase, gene3931: beta-glucanase, gene1280: 6-phospho-beta-galactosidase, gene2084: glucan endo-1,6-β-glucosidase, gene2945: alpha-glucosidase, gene0351: acetyl xylan esterase, gene1870: xylan 1,4-beta-xylosidase, gene1955: arabinoxylan arabinofuranohydrolase, gene2785: alpha-*N-*arabinofuranosidase, gene2795: arabinan endo-1,5-alpha-l-arabinosidase. The values represent the means of the three replicates with the standard deviation (SD). Asterisks represent significant differences from the glucose-containing medium (statistical significance: ** *P* < 0.01)

Expression profiles of selected *B. velezensis* LC1 lignocellulolytic enzymes genes were monitored in cultures grown on bamboo shoot powder compared to cultures utilizing glucose. Quantitative real-time PCR (qRT-PCR) investigation results of genes encoding endoglucanase (gene1950), beta-glucanase (gene3931), 6-phospho-beta-galactosidase (gene1280), glucan endo-1,6-β-glucosidase (gene2084), alpha-glucosidase (gene2945), acetyl xylan esterase (gene0351), xylan 1,4-beta-xylosidase (gene1870), arabinoxylan arabinofuranohydrolase (gene1955), alpha-*N*-arabinofuranosidase (gene2785), and arabinan endo-1,5-alpha-l-arabinosidase (gene2795) are shown in Fig. 4. Transcript levels of all the genes of interest were significantly up-regulated (at least *P* < 0.05) in BSP cultures compared to glucose cultures. It indicated that these genes involved in BSP degradation by *B. velezensis* LC1.

### Cellulose degradation efficiency and fermentation efficiency of bamboo shoots by *B. velezensis* LC1

**Fig. 5:**
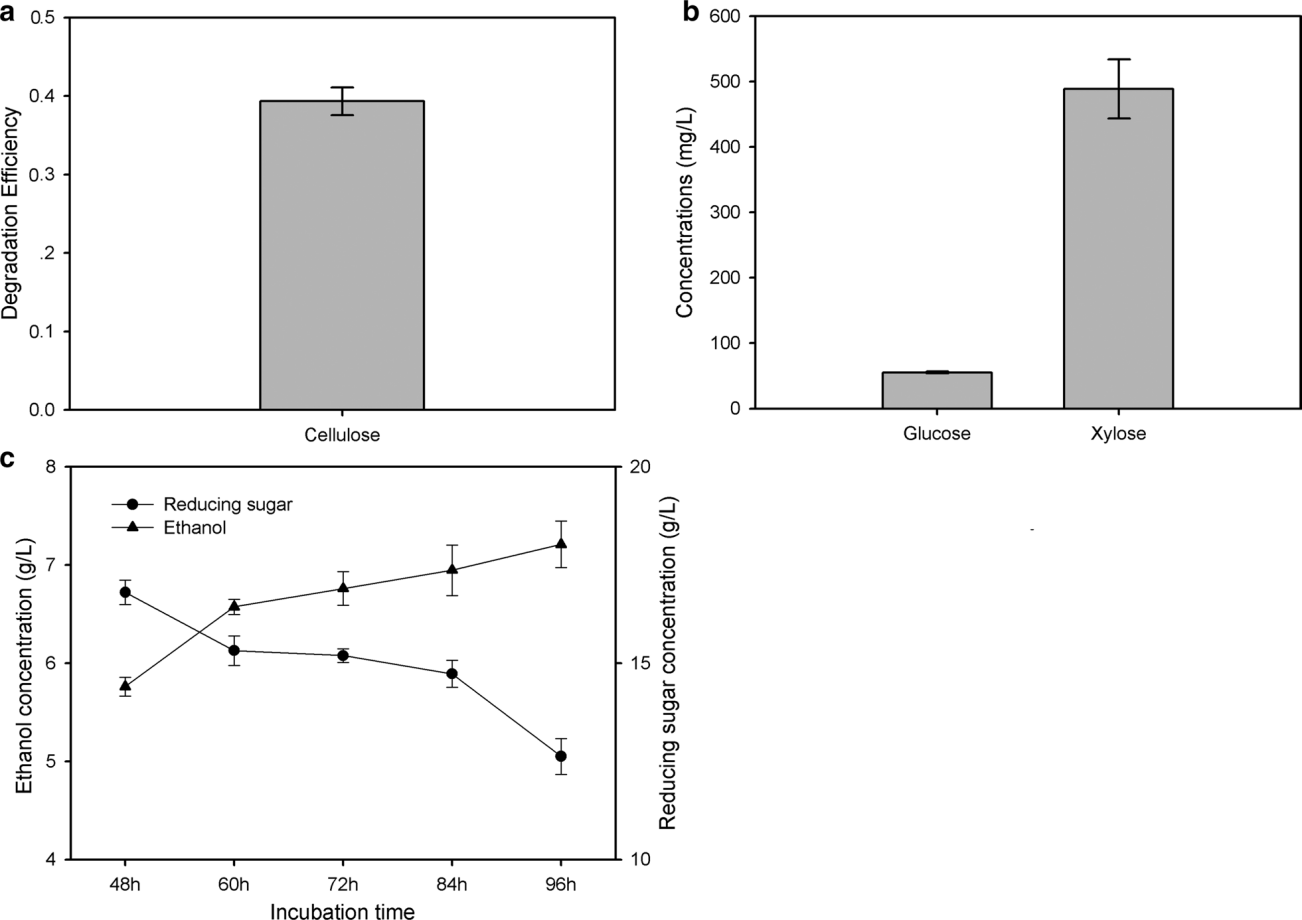
Cellulose-degrading efficiency and fermentation performance of BSP by *Bacillus velezensis* LC1. **a** Cellulose degradation efficiency. **b** Glucose and xylose contents of the hydrolysate. **c** Ethanol production at 48 h, 60 h, 72 h, 84 h and 96 h

Several previously reported genomes of *B. velezensis* strains contained genes encoding enzymes having lignocellulose-degrading potential [14–16]. However, lignocellulose-degrading abilities have not been completely verified in *B. velezensis* [12]. *B. velezensis* LC1, isolated from the intestine of *C. buqueti*, which was reported to be a microflora with lignocellulose-degrading ability, was prepared in a bamboo shoot powder (BSP) degradation assay [17]. We first determined the cellulose degradation efficiency to be 39.32% (Fig. 5a). Furthermore, we determined the glucose and xylose content in the degradation products to be 55.30 ± 1.40 mg/L and 488.81 ± 45.06 mg/L, respectively (Fig. 5b). The reducing sugars in the culture medium was mainly derived from the hydrolysis of cellulose and hemicellulose in BSPs, the reducing sugar content was determined to reflect the degree of conversion of lignocellulose. It indicated that the cellulose and hemicellulose of BSP were degraded with incubation with *B. velezensis* LC1.

Shimokawa et al. [43] reported that bamboo shoot was an excellent biomass stock for ethanol production due to its high saccharification efficiency. To determine the ethanol production capacity of *B. velezensis* LC1, a 6-day-treated hydrolysate was prepared for ethanol fermentation using glucose-fermenting *S. cerevisiae* and xylose-fermenting *E. coli* KO11. Ethanol production from BSP hydrolysate using *B. velezensis* LC1 is illustrated in Fig. 5c. The result showed that the ethanol yield continually increased during 48–96 h, and reached 7.21 ± 0.24 g/L at 96 h, while the reducing sugar continually decreased after incubation 48 h. This indicated that *B. velezensis* LC1 had a potent ability to convert lignocelluloses in bamboo shoot to ethanol.

## Conclusions

The genome of a gut symbiotic bacteria, *B. velezensis* LC1, was sequenced and analysed. The genome comprises a ring chromosome of 3,929,782 bp and has a GC content of 46.5%. A total of 136 CAZyme genes involved in lignocellulose degradation were annotated in the genome, and cellulose-degrading and ethanol-producing pathways were proposed. Moreover, the expression level of some CAZyme genes involved in cellulose and hemicellulose degradation, were up-regulated in bamboo shoot powder substrate. Moreover, a transcriptomic study would perform to identify the role of CAZyme genes in lignocellulose degradation as a future study. The cellulose degradation of bamboo shoot by the strain was 39.32%, and the hydrolysate was subjected to ethanol fermentation; the ethanol yield was 7.2 g/L at 96 h. This study suggests that *B. velezensis* LC1 could be used for bamboo lignocellulose degradation and bioconversion of lignocelluloses to ethanol.

## Methods

### Insect sample, isolation and identification of cellulolytic bacteria

*Cyrtotrachelus* *buqueti* specimens were sampled from the Muchuan County (E 103° 98′, N 28° 96′), China. Gut was extracted from individual insects and stored at 4 °C for isolation of bacteria. The gut was blended, homogenized and serially diluted (10^−1^ to 10^−9^), and inoculums of 10^−7^ to 10^−9^ dilution were plated on carboxymethyl cellulose (CMC) agar [44] for cellulolytic bacteria screening. Congo red dye was used to screen the cellulose-degrading bacteria as described by Teather and Wood [45]. Hydrolysis zone = clearance zone/colony diameter.

### Molecular characterization of bacterial isolate

The *16sRNA* V3–V4 region was considered for amplification for bacterial identification and amplified using bacterial primers (27F 5′-AGAGTTTGATCMTGGCTCAG-3′ and 1492R 5′-TACGGYTACCTTGTACGACTT-3′); moreover, F 5′-GCCCATATTTCCATTTCTCC-3′ and R 5′-GTGGTCGTTATGGAAATAAAGG-3′ were selected for amplification of the house-keeping gene *rpoB*. Thermocycling conditions were as follows: initial denaturation at 94 °C (2 min), followed by 30 cycles of denaturation at 94 °C (30 s), annealing at 55 °C (30 s) and extension at 72 °C (100 s), ultimately extending at 72 °C (2 min). The amplicons were checked by electrophoresis on a 1% agarose gel. MEGA5 was used to establish phylogenetic relationships among the obtained sequence and reference genes that were retrieved in NCBI GenBank through the neighbour-joining method.

### Genomic DNA extraction and genome sequencing

Genomic DNA of *B. velezensis* LC1 was extracted according to the CTAB method based on the protocol described by Lin [46], and it was purified using the Wizard Genomic DNA Purification Kit (Vazyme Biotech Co., Ltd, Nanjing, China). Whole-genome sequencing, performed by Shanghai Majorbio Bio-pharm Technology Co., Ltd (Shanghai, China), was fulfilled through PacBio systems with an average genome coverage of 100 × for the raw data [47]. HGAP 2.0 was used to filter and assemble reads to the scaffold.

### Function annotation of *B. velezensis* LC1

Glimmer 3.02 was used to predict coding DNA sequences (CDSs). A BLAST search was then conducted for CDSs in some widely used databases: NCBI non-redundant (NR) database, Swiss-Prot, COGs, Gene Ontology (GO) and KEGGs [48, 49]. GO, an important bioinformatics tool, unified expressions of gene and genetic products in all species [50]. KEGGs, a database resource to understand high-level functions, also could analyse metabolic pathways. Additionally, the CAZymes were identified, classified and annotated using CAZymes database (CAZyDB: http://www.cazy.org/).

### Quantitative real‑time PCR

The primers used for qRT-PCR in this study were performed in Additional file 7: Table S6. PCR was performed under following conditions: 10 min initial denaturation at 95 °C, 45 cycles of 5 s denaturation at 95 °C, 50–65 °C anneal for 30 s, and 30 s extension at 55 °C, finally 10 s extension at 95 °C. All experiments were performed three times and analysed by 2^− ΔΔCT^ Method. 16S rRNA was used as reference gene.

### Bamboo shoots degradation by *B. velezensis* LC1

To obtain fermentable sugar from bamboo shoots, *B. velezensis* LC1 was used to degrade the bamboo shoot powder (BSP), which was prepared as described by Luo et al. [17]. *B. velezensis* LC1 was cultured in liquid medium at a pH of 7.2, temperature of 37 °C and 200 rpm for 6 days. The culture medium comprised BSP 10 g/L, (NH4)_2_SO_4_ 2 g/L, K_2_HPO_4_ 1 g/L, KH_2_PO_4_ 1 g/L, MgSO_4_ 0.2 g/L, CaCl_2_ 0.1 g/L, FeSO_4_·7H_2_O 0.05 g/L and MnSO_4_·H_2_O 0.02 g/L. The reaction mixture was incubated at 100 °C for 30 min to terminate the reaction and centrifuged at 13,000 rpm for 10 min, and the hydrolysate-containing supernatant and deposit were collected separately. The obtained deposit was dried and weighed to determine the cellulose levels using the Van Soest method [51]. The hydrolysate-containing supernatant was used to determine the glucose and xylose contents according to the NREL methods [52]. The BSP hydrolysate supernatant was sterilized and stored at − 20 °C for ethanol fermentation.

### Fermentation

*Saccharomyces cerevisiae*, a glucose-fermenting strain, was pre-cultured in YPD at 30 °C for 24 h, and *E. coli* KO11, a xylose-fermenting strain, was cultured in LB at 37 °C for 24 h. *S. cerevisiae* (50 g/L) and *E. coli* KO11 (100 g/L) were prepared after centrifugation of pre-cultured cells. The initial cell concentrations were 0.33 g/L (*S. cerevisiae*) and 1.0 g/L (*E. coli* KO11) at the beginning of fermentation. 100 ml hydrolysate was used for ethanol fermentation in 250-ml serum bottles under anaerobic conditions. The fermentation was performed at 37 °C and 200 rpm for 96 h. After 48 h fermentation, ethanol production was monitored every 12 h. The ethanol concentration was determined via HPLC. All reactions were repeated three times.

## Supplementary information

**Additional file 1: Figure S1.** Cellulolytic activities of 4 isolates, PX9, PX10, PX11, and PX13, cultured on the CMC agar plate with congo red.

**Additional file 2: Table S1.** COG annotations of carbohydrate metabolism in genome of *B. velezensis* LC1.

**Additional file 3: Table S2.** GO annotations of carbohydrate metabolism in genome of *B. velezensis* LC1

**Additional file 4: Table S3.** KEGG Pathway classification of *B. velezensis* LC1.

**Additional file 5: Table S4.** Annotation of dioxygenase, monooxygenase, peroxidase, laccase and oxidoreductase genes identified in the genome of *B. velezensis* LC1.

**Additional file 6: Table S5.** CAZy annotation in the genome of *B. velezensis* LC1.

**Additional file 7: Table S6.** Primers used for RT-qPCR analysis.

## Data Availability

The sequence reads from this article have been deposited at the NCBI Sequence Read Archive under the accession PRJNA574012. The assembly data set supporting the results of this article has been deposited at GenBank under the accession CP044349. The version described in this paper is CP044349.

## Abbreviations

*B. velezensis*: *Bacillus velezensis*
GH: Glycoside hydrolase
GT: Glycosyl transferase
CE: Carbohydrate esterase
CBM: Carbohydrate-binding domain
PL: Polysaccharide lyase
AA: Auxiliary activities
CAZyme: Carbohydrate-active enzyme
NCBI: The National Center for Biotechnology Information
BSP: Bamboo shoot powder
YPD: Yeast extract peptone dextrose medium
DNS: 3,5-Dinitrosalicylic acid
CDS: Sequence coding for amino acids in protein

## References

[1] Naik SN, Goud VV, Rout PK, Dalai AK (2010). Production of first and second generation biofuels: a comprehensive review. Renew Sustain Energy Rev.

[2] Littlewood J, Wang L, Turnbull C, Murphy RJ (2013). Technoeconomic potential of bioethanol from bamboo in China. Biotechnol Biofuels.

[3] Ragauskas AJ, Beckham GT, Biddy MJ, Chandra R, Chen F, Davis MF (2014). Lignin valorization: improving lignin processing in the biorefinery. Science.

[4] Chen TY, Wen JL, Wang B, Wang HM, Liu CF, Sun RC (2017). Assessment of integrated process based on autohydrolysis and robust delignification process for enzymatic saccharification of bamboo. Bioresour Technol.

[5] Wi SG, Lee DS, Nguyen QA, Bae HJ (2017). Evaluation of biomass quality in short-rotation bamboo (*Phyllostachys pubescens*) for bioenergy products. Biotechnol Biofuels.

[6] Mansour AA, Da CA, Arnaud T, Lu-Chau TA, Fdz-Polanco M, Moreira MT (2016). Review of lignocellulolytic enzyme activity analyses and scale-down to microplate-based assays. Talanta.

[7] Zhang HY, Jackson TA (2008). Autochthonous bacterial fora indicated by PCR-DGGE of 16S rRNA gene fragments from the alimentary tract of *Costelytra zealandica* (Coleoptera: Scarabaeidae). J Appl Microbiol.

[8] de Gonzalo G, Colpa DI, Habib MH, Fraaije MW (2016). Bacterial enzymes involved in lignin degradation. J Biotechnol.

[9] Capolupo L, Faraco V (2016). Green methods of lignocellulose pretreatment for biorefinery development. Appl Microbiol Biotechnol.

[10] Gong G, Lee SM, Woo HM, Park TH, Um Y (2017). Influences of media compositions on characteristics of isolated bacteria exhibiting lignocellulolytic activities from various environmental sites. Appl Biochem Biotechnol.

[11] Khelil O, Choubane S, Cheba BA (2016). Polyphenols content of spent coffee grounds subjected to physico-chemical pretreatments influences lignocellulolytic enzymes production by *Bacillus* sp. R2. Bioresour Technol.

[12] Gong G, Kim S, Lee SM, Woo HM, Park TH, Um Y (2017). Complete genome sequence of *Bacillus* sp. 275, producing extracellular cellulolytic, xylanolytic and ligninolytic enzymes. J Biotechnol.

[13] Dunlap CA, Kim SJ, Kwon SW, Rooney AP (2016). *Bacillus velezensis* is not a later heterotypic synonym of *Bacillus amyloliquefaciens*; *Bacillus methylotrophicus*, *Bacillus amyloliquefaciens* sub sp. *plantarum* and ‘*Bacillus oryzicola*’ are later heterotypic synonyms of *Bacillus velezensis* based on phylogenomics. Int. J Syst Evol Microbiol.

[14] Kim SY, Song H, Sang MK, Weon HY, Song J (2017). The complete genome sequence of *Bacillus velezensis* strain GH1-13 reveals agriculturally beneficial properties and a unique plasmid. J Biotechnol.

[15] Liu G, Kong Y, Fan Y, Geng C, Peng D, Sun M (2017). Whole-genome sequencing of *Bacillus velezensis* LS69, a strain with a broad inhibitory spectrum against pathogenic bacteria. J Biotechnol.

[16] Niazi A, Manzoor S, Asari S, Bejai S, Meijer J, Bongcam-Rudloff E (2014). Genome analysis of *Bacillus amyloliquefaciens* subsp. *plantarum* UCMB5113: a rhizobacterium that improves plant growth and stress management. PLoS ONE.

[17] Luo CB, Li YQ, Chen Y, Fu C, Long WC, Xiao XM (2019). Bamboo lignocellulose degradation by gut symbiotic microbiota of the bamboo snout beetle *Cyrtotrachelus buqueti*. Biotechnol Biofuels.

[18] Potprommanee L, Wang XQ, Han YJ, Nyobe D, Peng YP, Huang Q (2017). Characterization of a thermophilic cellulase from *Geobacillus* sp. HTA426, an efficient cellulase-producer on alkali pre-treated of lignocellulosic biomass. PLoS ONE.

[19] Yadav S, Dubey SK (2018). Cellulose degradation potential of, *Paenibacillus lautus*, strain BHU3 and its whole genome sequence. Bioresour Technol.

[20] Dutta N, Mukhopadhyay A, Dasgupta AK, Chakrabarti K (2014). Improved production of reducing sugars from rice husk and rice straw using bacterial cellulase and xylanase activated with hydroxyapatite nanoparticles. Bioresour Technol.

[21] Chen L, Gu W, Xu H, Yang G, Shan X, Chen G (2018). Complete genome sequence of *Bacillus velezensis* 157 isolated from *Eucommia ulmoides* with pathogenic bacteria inhibiting and lignocellulolytic enzymes production by SSF. 3 Biotech.

[22] Zhang ZY, Raza MF, Zheng ZQ, Zhang XH, Dong XX, Zhang HY (2018). Complete genome sequence of *Bacillus velezensis* ZY-1-1 reveals the genetic basis for its hemicellulosic/cellulosic substrate-inducible xylanase and cellulase activities. 3 Biotech.

[23] Janeček Š, Gabriško M (2016). Remarkable evolutionary relatedness among the enzymes and proteins from the α–amylase family. Cell Mol Life Sci.

[24] Chaudhary N, Kumari I, Sandhu P, Ahmed M, Akhter Y (2016). Proteome scale census of major facilitator superfamily transporters in *Trichoderma reesei* using protein sequence and structure based classification enhanced ranking. Gene.

[25] Madej MG, Sun L, Yan N, Kaback HR (2014). Functional architecture of MFS d-glucose transporters. Proc Natl Acad Sci USA.

[26] Wang C, Dong D, Wang H, Müller K, Qin Y, Wang H (2016). Metagenomic analysis of microbial consortia enriched from compost: new insights into the role of Actinobacteria in lignocellulose decomposition. Biotechnol Biofuels.

[27] Rashid N, Imanaka H, Fukui T, Atomi H, Imanaka T (2004). Presence of a novel phosphopentomutase and a 2-deoxyribose 5-phosphate aldolase reveals a metabolic link between pentoses and central carbon metabolism in the hyperthermophilic archaeon *Thermococcus kodakaraensis*. J Bacteriol.

[28] Roth C, Weizenmann N, Bexten N, Saenger W, Zimmermann W, Maier T (2017). Amylose recognition and ring-size determination of amylomaltase. Sci Adv.

[29] Pandey S, Kushwa J, Tiwari R, Kumar R, Somvanshi VS, Nain L (2014). Cloning and expression of β-1, 4-endoglucanase gene from *Bacillus subtilis* isolated from soil long term irrigated with effluents of paper and pulp mill. Microbiol Res.

[30] Graebin NG, Schoffer Jda N, Andrades D, Hertz PF, Ayub MA, Rodrigues RC (2016). Immobilization of glycoside hydrolase families GH1, GH13, and GH70: state of the art and perspectives. Molecules.

[31] Bezzate S, Steinmetz M, Aymerich S (1994). Cloning, sequencing, and disruption of a levanase gene of *Bacillus polymyxa* CF43. J Bacteriol.

[32] Romero AM, Mateo JJ, Maicas S (2012). Characterization of an ethanol-tolerant 1,4-beta-xylosidase produced by *Pichia membranifaciens*. Lett Appl Microbiol.

[33] Vanholme B, Jacob J, Cannoot B, Gheysen G, Haegeman A (2009). Arabinogalactan endo-1,4-β-galactosidase: a putative plant cell wall-degrading enzyme of plant-parasitic nematodes. Nematology.

[34] Onilude AA, Fadahunsi IF, Antia UE, Garuba EO, Inuwa M (2013). Characterization of crude alkaline β-mannosidase produced by *Bacillus* sp. 3A isolated from degraded palm kernel cake. J Inorg Biochem.

[35] Inoue Y, Yasutake N, Oshima Y, Yamamoto Y, Tomita T, Miyoshi S (2001). Cloning of the maltose phosphorylase gene from *Bacillus* sp. strain RK-1 and efficient production of the cloned gene and the trehalose phosphorylase gene from *Bacillus stearothermophilus* SK-1 in *Bacillus subtilis*. Biosci Biotechnol Biochem.

[36] Zhang J, Siika-Aho M, Tenkanen M, Viikari L (2011). The role of acetyl xylan esterase in the solubilization of xylan and enzymatic hydrolysis of wheat straw and giant reed. Biotechnol Biofuels.

[37] Mamo G, Hatti-Kaul R, Bo M (2006). A thermostable alkaline active endo-β-1-4-xylanase from *Bacillus halodurans* S7: purification and characterization. Enzyme Microbial Technol.

[38] Zhao Z, Liu H, Wang C, Xu JR (2014). Erratum to: comparative analysis of fungal genomes reveals different plant cell wall degrading capacity in fungi. BMC Genom.

[39] Biely P (2012). Microbial carbohydrate esterases deacetylating plant polysaccharides. Biotechnol Adv.

[40] See-Too WS, Chua KO, Lim YL, Chen JW, Convey P, Mohd Mohidin TB (2017). Complete genome sequence of *Planococcus donghaensis* JH1(T), a pectin-degrading bacterium. J Biotechnol.

[41] van den Heuvel RH, Fraaije MW, Mattevi A, van Berkel WJ (2002). Structure, function and redesign of vanillyl-alcohol oxidase. Int Congress Ser.

[42] Levasseur A, Drula E, Lombard V, Coutinho PM, Henrissat B (2013). Expansion of the enzymatic repertoire of the CAZy database to integrate auxiliary redox enzymes. Biotechnol Biofuels.

[43] Shimokawa T, Ishida M, Yoshida S, Nojiri M (2009). Effects of growth stage on enzymatic saccharification and simultaneous saccharification and fermentation of bamboo shoots for bioethanol production. Bioresour Technol.

[44] Mondéjar LR, Zühlke D, Becher D, Riedel K, Baldrian P (2016). Cellulose and hemicellulose decomposition by forest soil bacteria proceeds by the action of structurally variable enzymatic systems. Sci Rep.

[45] Teather RM, Wood PJ (1959). Use of Congo red-polysaccharide interactions in enumeration and characterization of cellulolytic bacteria from bovine rumen. Appl Environ Microbiol.

[46] Lin T, Zhu G, Zhang J, Xu X, Yu Q, Zheng Z (2014). Genomic analyses provide insights into the history of tomato breeding. Nat Genet.

[47] Westbrook CJ, Karl JA, Wiseman RW, Mate S, Koroleva G, Garcia K (2015). No assembly required: full-length MHC class I allele discovery by PacBio circular consensus sequencing. Hum Immunol.

[48] Gao XY, Zhi XY, Li HW, Klenk HP, Li WJ (2014). Comparative genomics of the bacterial genus *Streptococcus* illuminates evolutionary implications of species groups. PLoS ONE.

[49] Sun C, Fu GY, Zhang CY, Hu J, Xu L, Wang RJ (2016). Isolation and complete genome sequence of *Algibacter alginolytic*a sp. nov., a novel seaweed-degrading Bacteroidetes bacterium with diverse putative polysaccharide utilization Loci. Appl Environ Microbiol.

[50] Huntley RP, Sawford T, Mutowo-Meullenet P, Shypitsyna A, Bonilla C, Martin MJ (2015). The GOA database: gene ontology annotation updates for 2015. Nucleic Acids Res.

[51] Van Soest PJ, Robertson JB, Lewis BA (1991). Methods for dietary fiber, neutral detergent fiber, and nonstarch polysaccharides in relation to animal nutrition. J Dairy Sci.

[52] Sluiter A, Hames B, Ruiz R, Scarlata C, Sluiter J, Templeton D (2012). Determination of structural carbohydrates and lignin in biomass. version 2012.

