# Supplementary figures and images for: Genome sequencing of gut symbiotic Bacillus velezensis LC1 for bioethanol production from bamboo shoots
Source: Biotechnol Biofuels. 2020 Feb 28;13:34. doi: 10.1186/s13068-020-1671-9 (PMC7048129; doi:10.1186/s13068-020-1671-9)

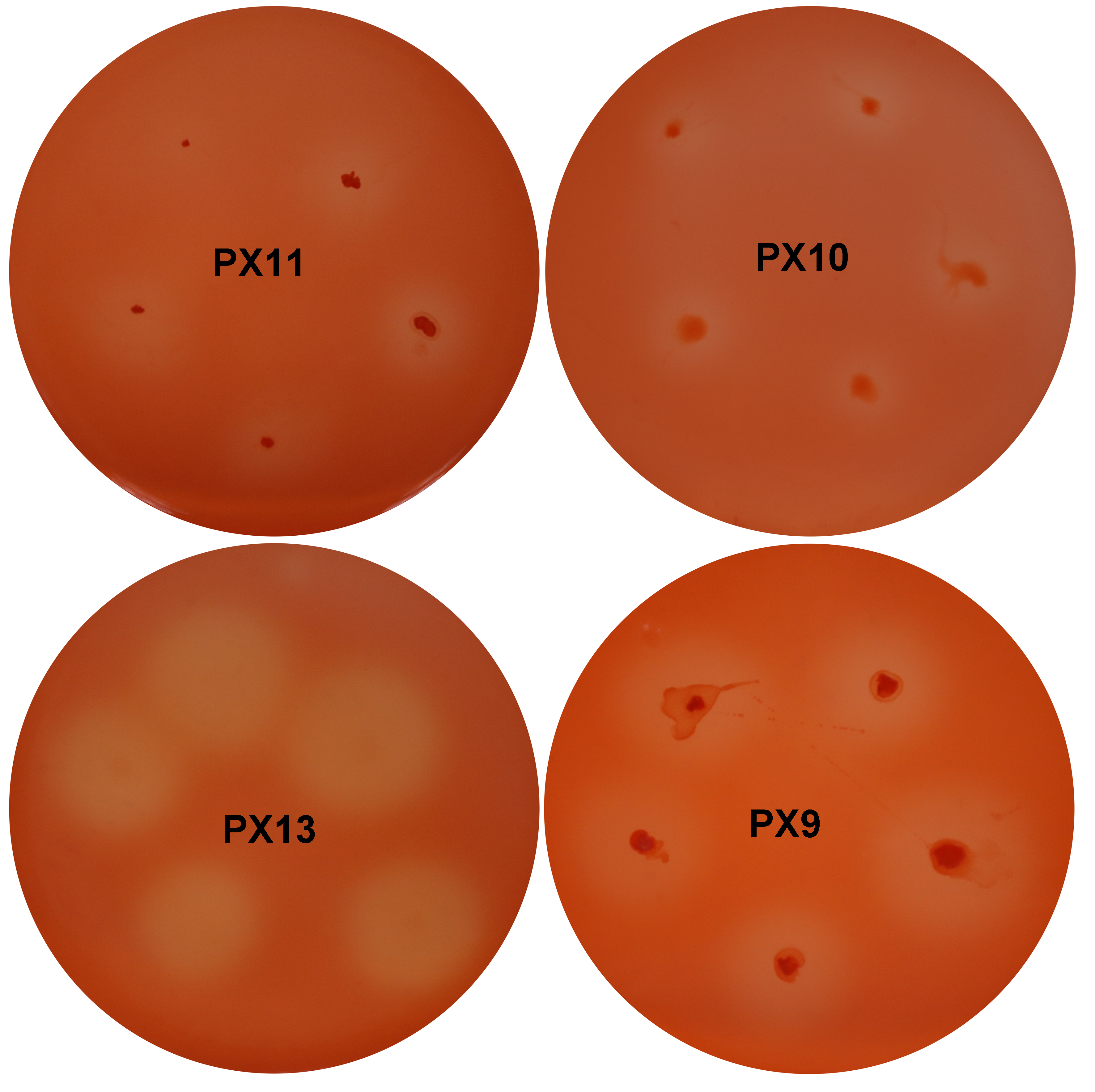

Supplement: Supplementary file 1 — Additional file 1: Figure S1. Cellulolytic activities of 4 isolates, PX9, PX10, PX11, and PX13, cultured on the CMC agar plate with congo red. [file 13068_2020_1671_MOESM1_ESM.tif]
